# Intraoperative low tidal volume ventilation and the risk of ICD-10 coded delirium and the use for antipsychotic medications

**DOI:** 10.1186/s12871-022-01689-3

**Published:** 2022-05-16

**Authors:** Dharshi Karalapillai, Laurence Weinberg, Ary Serpa Neto, Philip J. Peyton, Louise Ellard, Raymond Hu, Brett Pearce, Chong Tan, David Story, Mark O’Donnell, Patrick Hamilton, Chad Oughton, Jonathan Galtieri, Sree Appu, Anthony Wilson, Glenn Eastwood, Rinaldo Bellomo, Daryl A. Jones

**Affiliations:** 1 Department of Anaesthesia, Austin Hospital, Melbourne, VIC Australia; 2 Department of Intensive Care, Austin Hospital, Melbourne, VIC Australia; 3 Department of Critical Care, The University of Melbourne, Melbourne, VIC Australia; 4 Department of Surgery, University of Melbourne, Melbourne, VIC Australia; 5 Australian and New Zealand Intensive Care Research Centre, School of Public Health and Preventive Medicine, Monash University, Melbourne, VIC Australia; 6 Data Analytics Research and Evaluation (DARE) Centre, University of Melbourne, Melbourne, VIC Australia; 7 Department of Critical Care Medicine, Hospital Israelita Albert Einstein, São Paulo, Brazil; 8 Department of Surgery, Austin Hospital, Melbourne, VIC Australia

**Keywords:** Delirium, Postoperative, Surgery, Tidal volume

## Abstract

**Background:**

Low tidal volume (V_T_) ventilation and its associated increase in arterial carbon dioxide (PaCO_2_) may affect postoperative neurologic function. We aimed to test the hypothesis that intraoperative low V_T_ ventilation affect the incidence of postoperative ICD-10 coded delirium and/or the need for antipsychotic medications.

**Methods:**

This is a post-hoc analysis of a large randomized controlled trial evaluating low vs. conventional V_T_ ventilation during major non-cardiothoracic, non-intracranial surgery. The primary outcome was the incidence of ICD-10 delirium and/or the use of antipsychotic medications during hospital stay, and the absolute difference with its 95% confidence interval (CI) was calculated.

**Results:**

We studied 1206 patients (median age of 64 [55–72] years, 59.0% males, median ARISCAT of 26 [19–37], and 47.6% of ASA 3). ICD-10 coded delirium and /or antipsychotic medication use was diagnosed in 11.2% with similar incidence between low and conventional V_T_ ventilation (11.1% vs. 11.3%; absolute difference, -0.24 [95%CI, -3.82 to 3.32]; *p* = 0.894). There was no interaction between allocation group and type of surgery.

**Conclusion:**

In adult patients undergoing major surgery, low V_T_ ventilation was not associated with increased risk of ICD-10 delirium and/or the use of antipsychotic medications during hospital stay.

**Trial registration:**

ANZCTR Identifier: ACTRN12614000790640.

**Supplementary Information:**

The online version contains supplementary material available at 10.1186/s12871-022-01689-3.

## Background

Low tidal volume (V_T_) ventilation during major surgery is associated with increased arterial carbon dioxide tension (PaCO_2_) [1]. The effect of an increased PaCO_2_ on neurologic function is likely complex and both favourable and unfavourable neurologic effects have been described in different clinical settings [2–7]. Similarly, the association of increased intraoperative PaCO_2_ levels and the incidence of post-operative delirium (POD) is unclear, and previous small observational studies have yielded mixed results [8–12]. Given that POD is common, associated with poor outcomes and may be partly preventable, identifying strategies that reduce its incidence by targeting potential modifiable risk factors such as PaCO_2_ appears desirable [13–17].

Recently, a large randomised clinical trial showed that the use of low vs. conventional V_T_ ventilation during major surgery did not change the incidence of post-operative pulmonary complications (PPC) [1]. However, low V_T_ was associated with significantly higher intraoperative PaCO_2_. This effect provides a unique opportunity to assess the impact of low V_T_ ventilation and intra-operative PaCO_2_ levels on the incidence of postoperative ICD 10 (International Classification of Diseases 10^th^ revision) coded delirium and/or the use of antipsychotic medications. Accordingly, we performed a post-hoc analysis of this trial to test the hypothesis that low V_T_ ventilation during surgery would be associated with an increased incidence of ICD-10 coded delirium and/or the use of antipsychotic medications in adult patients undergoing major surgery.

## Methods

### Study design

This was a post-hoc analysis of an investigator-initiated, assessor-blinded, single-centre, randomized clinical trial. The protocol and statistical analysis plan [18], and the primary trial have been published [1]. The local human research ethics committee of the Austin Hospital approved the study (HREC approval number HREC/14/Austin260). Written informed consent was obtained from all participating patients. This study was performed in accordance with the Declaration of Helsinki. The primary trial was registered with the ANZCA clinical trials network (ACTRN12614000790640).

### Patients

Patients were included in the primary trial if they were older than 40 years of age, scheduled to have major surgery of expected duration > 2 h, and planned to have invasive arterial pressure monitoring as part of their routine care. Patients were excluded if they were pregnant, scheduled to have cardiac, thoracic or intracranial neurological surgery, or if they had been previously enrolled in the trial [1, 18].

### Details of ventilation and timing of data collection

As described in the study protocol and in our primary trial, all patients received volume-controlled ventilation with an applied positive end expiratory pressure (PEEP) of 5 cmH_2_O. Immediately after randomization, patients were assigned to receive either low V_T_ (6 mL/kg predicted body weight [PBW]) or a conventional V_T_ (10 mL/kg PBW) ventilation. PBW was calculated as 50 + 0.91*(height [cm] – 152.4) for males and 45.5 + 0.91*(height [cm] – 152.4) for females. The V_T_ and PEEP were maintained for the whole duration of the surgical procedure [1, 18].

As described in the study protocol and in our primary trial, all cases were performed under the supervision or direct care of a specialist anaesthetist. Participants underwent intravenous induction, neuromuscular blockade and endotracheal intubation, and a volatile agent was used to maintain anaesthesia. The primary trial from which this sub-study was derived investigated a single isolated change in set tidal volume and its effects on outcomes. This was designed as a pragmatic trial and all other aspects of clinical care including targets for End tidal carbon dioxide (ETCO2), PaCO_2_ and oxygenation (SpO_2_ and PaO_2_) were at the discretion of the treating anaesthetist. In addition, the inspired fraction of oxygen (FiO_2_), respiratory rate, anaesthesia technique, fluid management, use of vasoactive drugs, analgesia plan, use of prophylactic antibiotics and anti-emetics agents were administered at the discretion of the treating anaesthesiologist [1, 18].

As part of the study protocol of our primary trial, the treating anaesthetists obtained an arterial blood gas (ABG) 15 min after induction of anaesthesia (‘after induction’), and ‘pre-emergence’ of anaesthesia. These time points were chosen to reflect the PaCO_2_ during the maintenance phase of anaesthesia. The results of the ABG were presented to the treating anaesthetist by nurses and subsequent management was dictated according to their clinical judgment. Postoperatively in the post-anaesthesia care unit (PACU), a third ABG was obtained approximately 15 min after the patient’s arrival in the PACU. Regarding the measurement of the PaCO_2,_ this was obtained using an ABL 800 Blood gas analyser (Radiometer, Copenhagen, Denmark). ABG variables include partial pressure of oxygen (PaO_2_), PaCO_2_, pH, bicarbonate concentration, base excess, lactate, haemoglobin concentration (Hb), and electrolytes such as sodium and potassium concentrations [1, 18].

### Details of data collected

As described in our primary trial, a standardized case report form was used for data collection. Intraoperatively, all ventilatory data and vital signs were collected prospectively as the lowest and/or highest values during the procedure. The research staff collected all data directly from the clinical chart source data. Until postoperative day 7 or hospital discharge (whichever came first), all patients were assessed daily by the trial’s research team. Research staff blinded to the intraoperative intervention collected information regarding the clinical outcomes. After the first seven days (if the patient was still in hospital), additional data were retrieved from the electronic medical record [1].

### Outcomes

The primary outcome was the incidence of ICD-10 coded delirium during hospital stay and/or the need for a new prescription (i.e., not a pre-operative medication) of any dose of the following antipsychotic medications: olanzapine, quetiapine, risperidone, haloperidol and/or diazepam via any route of administration). These medications were chosen after an internal audit at our centre identified these agents as the only agents used for the pharmacologic treatment of delirium. Pharmacologic prophylaxis for delirium is not used in our institution and is reserved for treatment of POD refractory to non-pharmacologic measures. The use of other antipsychotics or benzodiazepines for the management of POD would therefore be considered rare.

Postoperative delirium was diagnosed by the treating clinical team and ICD -10 coded delirium was identified by hospital coders during the patient’s hospital stay (ICD-10 codes: F05.0 ‘‘Delirium not superimposed on dementia, so described”; F05.1 ‘‘Delirium superimposed on dementia”; F05.8 ‘‘Other delirium”; and F05.9 ‘‘Delirium, unspecified”). Assessors were blinded to the purpose of the study. In, addition, given the possibility of insufficient information to allow ICD-10 coded delirium to be identified, data from the electronic prescription system for any newly prescribed antipsychotic drugs (as described above) and for diazepam, and the timing, number and total dose administered were extracted.

The key secondary outcome was the incidence of delirium as described above but excluding the use of diazepam from the definition. Other secondary outcomes include the number of times that new antipsychotic or anxiolytic drugs were administered during hospital stay, and the total dose of antipsychotic drugs used during hospital stay (calculated as haloperidol equivalents) [19, 20]. Regarding benzodiazepines, their use for the management of delirium in our region (in the absence of an alcohol withdrawal) is generally discouraged and to our knowledge not practiced. This was included the use of benzodiazepines as a secondary outcome only. The use of other benzodiazepines (other than diazepam) would be considered very unusual for the management of post-operative delirium in our institution.

### Statistical analysis

Categorical variables are reported as counts and percentages and compared with Fisher exact tests, and continuous variables as median (interquartile range) and compared with Wilcoxon rank-sum test. Patients were analysed according to the group they were randomized in the original trial, and the analysis dataset included all patients who were randomized and had general anaesthesia for eligible surgery. Because the amount of missing data for the primary outcome was small, only a complete case analysis was carried out and no assumption for missing data was made.

The incidence of ICD-10 coded delirium and /or the use of antipsychotic medications during hospital stay was reported in each arm of the original trial, and the risk difference with its 95% confidence interval was calculated from an unadjusted generalized linear model considering a binomial distribution with an identity link. The difference in the number of times that an anxiolytic or an antipsychotic was used, and the total dose of antipsychotic drugs was calculated as a median difference from a quantile model considering a Τ = 0.50 and an interior point algorithm. *P* values were extracted after 1,000 bootstrap samplings. All models were not adjusted for confounders.

As a sensitivity analysis, and to further understand the findings, an interaction between the treatment allocation and type of surgery (open vs. laparoscopic) was assessed. A two–sided *p* value < 0.05 was considered as evidence of statistical significance. All analyses were performed using R software, version 4.0.3 (R Core Team).

## Results

### Patients

All 1206 patients included in the original trial, recruited between February 2014 and February 2019 were included in the present analysis. From this group, 614 (50.9%) were randomized to the low V_T_ group and 592 (49.1%) to conventional V_T_ group. Median age of the included patient was 64 (55—72), 59.0% of the patients were male, and median ARISCAT score was 26 (19—37). Hypertension was the most prevalent co-morbidity (52.1%) followed by obesity (37.3%), diabetes (20.3%) and smoking (17.3%) (Table 1). The majority of the patients underwent abdominal surgery (56.1%) and, of these, 48.2% were laparoscopic. Median duration of surgery was 187 (136—257) minutes. All baseline characteristics were similar between the two groups (Table 1).

**Table 1:** Baseline characteristics of the included patients

	**Low Tidal Volume** **(*n* = 614)**	**Conventional Tidal Volume** **(*n* = 592)**
Age, years	65.0 (54.0 – 72.0)	64.0 (55.0 – 72.0)
Male gender	366 (59.6)	346 (58.4)
Body weight, kg		
Actual	80.0 (68.0 – 95.0)	80.5 (70.5 – 94.0)
Predicted	63.3 (56.0 – 70.6)	64.2 (55.1 – 70.6)
Body mass index, kg/m^2^	27.9 (24.4 – 32.5)	28.1 (25.1 – 32.0)
ARISCAT risk score	26.0 (19.0 – 37.0)	26.0 (19.0 – 35.8)
Low	193 (34.8)	196 (37.8)
Moderate	324 (58.5)	282 (54.4)
High	37 (6.7)	40 (7.7)
Preoperative SpO_2_, %	97.0 (96.0 – 98.0)	97.0 (96.0 – 98.0)
Preoperative HCO_3_, mmol/L	26.0 (24.0 – 27.0)	26.0 (24.0 – 28.0)
Preoperative haemoglobin, g/dL	138.0 (127.0 – 149.0)	138.0 (124.0 – 149.0)
Preoperative creatinine, mg/dL	0.88 (0.74 – 1.06)	0.87 (0.75 – 1.06)
Co-morbidities		
Diabetes mellitus	119 (19.4)	126 (21.3)
Hypertension	301 (49.1)	327 (55.3)
Coronary artery disease	93 (15.2)	100 (16.9)
Chronic renal disease	56 (9.1)	67 (11.3)
Chronic liver disease	48 (7.8)	52 (8.8)
Current smoker	100 (16.3)	109 (18.4)
COPD	62 (10.1)	65 (11.0)
Asthma	66 (10.7)	68 (11.5)
Interstitial lung disease	8 (1.3)	2 (0.3)
Bronchiectasis	1 (0.2)	1 (0.2)
Obstructive sleep apnoea	59 (9.6)	63 (10.6)
Obesity^a^	225 (37.8)	207 (36.8)
Recent LRTI	8 (1.3)	8 (1.4)
Type of Surgery		
Abdominal	348 (56.7)	333 (56.3)
Laparoscopic	158 / 348 (45.4)	170 / 333 (51.1)
General	6 (1.0)	2 (0.3)
Ear, nose and throat	17 (2.8)	13 (2.2)
Orthopaedic	43 (7.0)	46 (7.8)
Plastic	31 (5.0)	36 (6.1)
Spine	125 (20.4)	120 (20.3)
Vascular	29 (4.7)	28 (4.7)
Others	15 (2.4)	13 (2.2)
Duration of surgery, minutes	189.5 (135.0 – 267.5)	185.0 (140.5 – 249.5)

Data are presented as median (quartile 25—quartile 75) or N (%)

*ARISCAT* Assess Respiratory Risk in Surgical Patients in Catalonia*, COPD* chronic obstructive pulmonary disease*, HCO*_*3*_ bicarbonate*, LRTI* lower respiratory tract infection*, SpO*_*2*_ pulse oximetry

^a^ defined as BMI > 30 kg/m^2^

Within the first seven postoperative days, 38.6% of the patients developed postoperative pulmonary complications, 9.6% developed acute kidney injury and 4.7% were admitted unexpectedly to the ICU (eTable 1 in the Online Supplement). In-hospital mortality rate was 1.2%. All clinical outcomes were similar between the allocation groups.

### PaCO_2_ during surgery

**Fig. 1:**
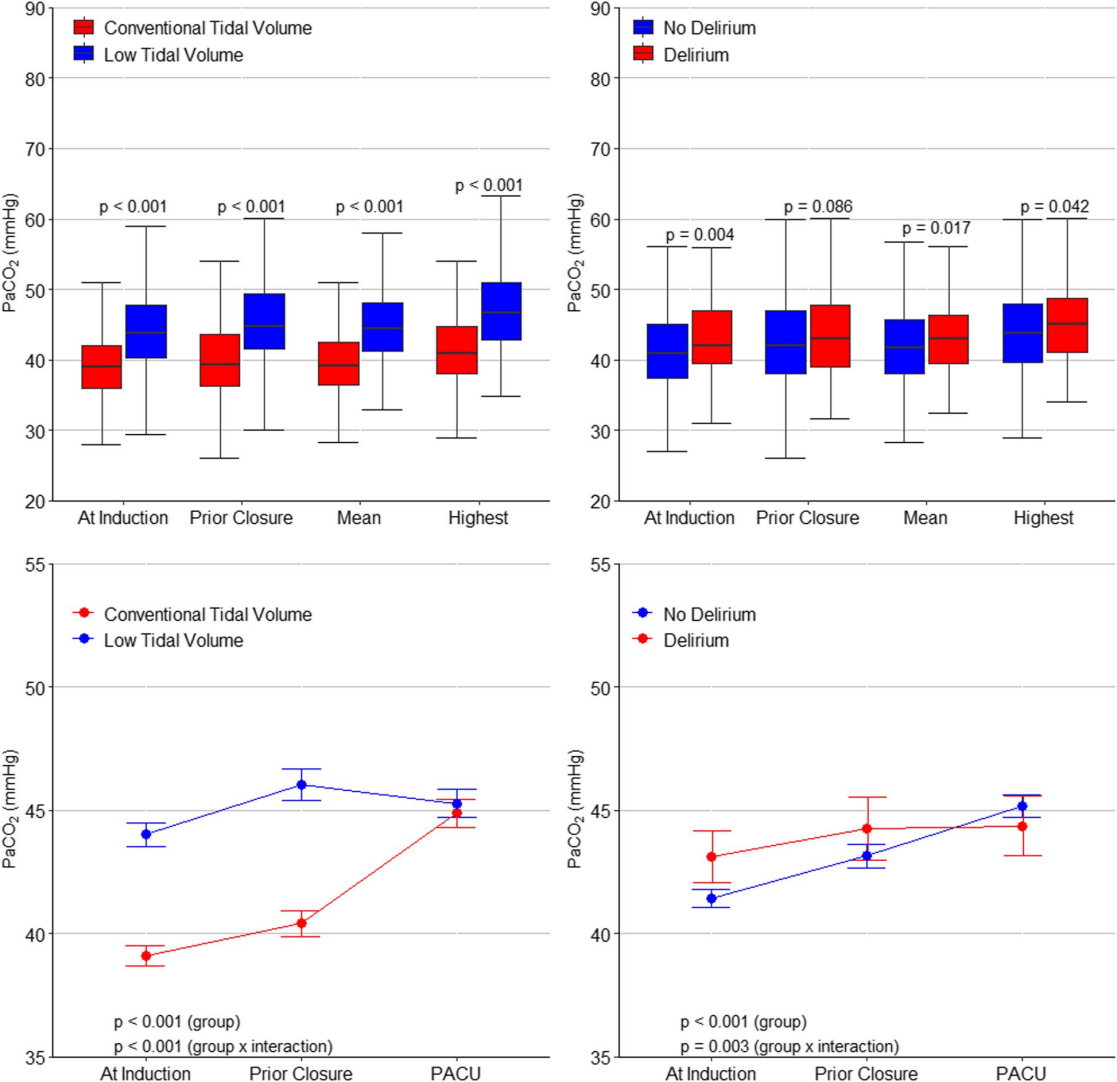
PaCO_2_ Levels According to the Allocation Group, and Development of Delirium. Upper panels, boxplots with *p* values calculated with Wilcoxon rank-sum test. Bottom panels, circles are mean and error bars 95% confidence interval. *P* from a mixed-effect linear model with an interaction of group and time (as a continuous variable) and with patients as random effects

The distribution of mean PaCO_2_ during surgery according to allocation group and to presence or absence of POD is shown in eFigure 1 in Online Supplement. The PaCO_2_ levels were consistently higher in patients in the low V_T_ group and in patients who developed POD (Fig. 1).

### Outcomes

The incidence of ICD-10 coded delirium and/or the use of antipsychotic medications was similar in patients in the low V_T_ group compared to conventional V_T_ group (11.1% vs. 11.3%; absolute difference, -0.24 [95% CI, -3.82 to 3.32]; *p* = 0.894) (Table 2). This finding was sustained after the use of diazepam was removed from the definition of antipsychotic medications.

**Table 2:** Primary and Secondary Outcomes According to the Allocation Group

	**Low Tidal Volume** **(*n* = 614)**	**Conventional Tidal Volume** **(*n* = 592)**	**Absolute Difference** **(95% CI)**	***p* value**
**Primary outcome**				
Delirium during hospital stay	68 (11.1)	67 (11.3)	-0.24 (-3.82 to 3.32)	0.894
**Key secondary outcome**				
Delirium during hospital stay^a^	68 (11.1)	67 (11.3)	-0.24 (-3.82 to 3.32)	0.894
**Secondary outcomes**				
Number of doses of anxiolytic	3.5 (2.0 – 7.5)	3.0 (1.0 – 10.0)	0.66 (-1.58 to 2.90)	0.562
Number of doses of antipsychotic	3.0 (2.0 – 6.0)	4.0 (1.5 – 10.5)	-1.00 (-4.95 to 2.95)	0.622
Total dose of antipsychotic	9.4 (3.0 – 20.9)	4.0 (2.5 – 30.0)	5.98 (-7.15 to 19.12)	0.376

Data are presented as median (quartile 25—quartile 75) or N (%)

Absolute difference is risk difference for primary and key secondary outcomes and median difference for secondary outcomes

^a^ Excluding diazepam from the definition

The number of doses of anxiolytic (3 [2–7] vs. 3 [1–10]; median difference, -0.66 [95%CI, -1.58 to 2.90]; *p* = 0.562) and antipsychotic medications (3 [2–6] vs. 3 [1–10]; median difference, -1.00 [95%CI, -4.95 to 2.95]; *p* = 0.622), and the total dose of antipsychotics (9 [3–21] vs. 4 [2–30]; median difference, 5.98 [95%CI, -7.15 to 19.12]; *p* = 0.376) were similar between the allocation groups (Table 2).

### Sensitivity analysis

Comparison of the allocation groups according to the type of surgery is shown in eTable 2 in the Online Supplement. There was no interaction between the allocation group and the type of surgery for the outcomes assessed (Table 3).

**Table 3:** Primary and Secondary Outcomes According to the Allocation Group and to the Type of Surgery

	**Laparoscopic Surgery**				**Open Surgery**				***p* for Interaction**
	**Low Tidal Volume** **(*n* = 158)**	**Conventional Tidal Volume** **(*n* = 170)**	**Absolute Difference** **(95% CI)**	***p* value**	**Low Tidal Volume** **(*n* = 456)**	**Conventional Tidal Volume** **(*n* = 422)**	**Absolute Difference** **(95% CI)**	***p* value**	
**Primary outcome**									
Delirium during hospital stay	13 (8.2)	15 (8.8)	-0.60 (-6.74 to 5.61)	0.847	55 (12.1)	52 (12.3)	-0.26 (-4.63 to 4.07)	0.906	0.930
**Key secondary outcome**									
Delirium during hospital stay	13 (8.2)	15 (8.8)	-0.60 (-6.74 to 5.61)	0.847	55 (12.1)	52 (12.3)	-0.26 (-4.63 to 4.07)	0.906	0.930
**Secondary outcomes**									
Number of doses of anxiolytic	3.0 (1.0—4.0)	2.5 (1.0—3.2)	0.19 (-2.82 to 3.19)	0.904	4.0 (2.0—8.0)	4.0 (1.0—11.0)	-0.00 (-3.16 to 3.16)	0.999	0.864
Number of doses of antipsychotic	3.0 (1.8—3.8)	2.0 (1.2—2.8)	1.00 (-3.62 to 5.62)	0.679	4.0 (2.0—10.5)	6.0 (3.0—11.0)	-2.00 (-8.70 to 4.70)	0.562	0.526
Total dose of antipsychotic	9.0 (3.8—13.4)	2.5 (2.0—3.8)	3.20 (-7.09 to 13.50)	0.552	9.4 (2.2—23.5)	12.0 (3.0—48.0)	-1.96 (-29.20 to 25.27)	0.888	0.554

Data are presented as median (quartile 25—quartile 75) or N (%)

Absolute difference is risk difference for primary and key secondary outcomes and median difference for secondary outcomes

## Discussion

### Summary of findings

We conducted a post-hoc analysis of a large randomized clinical trial to evaluate the association between intraoperative low V_T_ ventilation and ICD-10 coded delirium and/or the use of antipsychotic medications. The findings suggest that whilst low V_T_ ventilation was associated with increased intraoperative PaCO_2_, it was not associated with an increased incidence of ICD-coded delirium and/or antipsychotic medications use compared to the conventional V_T_ ventilation. In addition, there was no interaction between tidal volume size and the type of surgery on ICD-coded delirium and /or antipsychotic medications use.

### Comparison with previous studies

To our knowledge this is the first study based on clinical data obtained from a large randomized clinical trial to evaluate the association between intraoperative low V_T_ ventilation and the risk of ICD-10 coded delirium and the use of antipsychotic medications. Previous clinical trials of low V_T_ ventilation have not reported the effect of low V_T_ ventilation on cognitive function [21].

Multiple studies have attempted identify risk factors for post-operative delirium (POD), but there is little information on the possible impact of intraoperative ventilation and PaCO_2_ levels on development of POD [22–25]. PaCO_2_ is a fundamental determinant of cerebral blood flow, cerebral metabolism and intracranial pressure, which suggests a biologically plausible mechanism for an effect on cognitive function [2, 3]. However, to the date, studies assessing the association between hypercapnia and POD have yielded inconsistent and conflicting results [8–12]. Furthermore a recent observational study suggested that avoidance of end-tidal hypocapnia may be associated with a reduced risk of POD [26].

### Implications for clinical practice and further research

Our study implies that low V_T_ is associated with increase in intraoperative PaCO_2_ but not with an increased incidence of ICD-10 coded delirium and/or the use of antipsychotic medications. Moreover, they imply that no interaction with the type of surgery is present.

### Strengths and limitations

This study is the largest study assessing the effect of low V_T_ ventilation during major surgery and its impact on ICD-10 coded delirium and/or the use for antipsychotic medications. Moreover, to our knowledge, it is the only study based on serial ABG analysis with prospectively collected data on more than 3000 ABG analyses. Such systematic data collection allowed detailed analysis and control of intraoperative PaCO_2_. Also, the assessment of outcomes was blinded to treatment allocation, attenuating ascertainment bias. In addition, we selected patients with surgery expected to last at least 2 h, to increase the ability to identify the putative effect of the mechanical ventilation strategy. Furthermore, multiple types of surgery were included which increased the generalizability of our findings.

We acknowledge several limitations. First, this is a post hoc analysis of a clinical trial, thus no causal relationship can be determined or inferred. Second, it is a single centre study with all the inherent limitations of such studies. However, it did include a diverse range of patients and surgeries and involved more than 140 anaesthetists. Third we acknowledge that a major weakness of our study relates to the identification of delirium by coders which may be of limited accuracy. ICD-10 coding for delirium lacks sensitivity as previously reported [27, 28]. However, this method has strong specificity (up to 99% in postoperative patients) [27, 28]. As such, although many cases of delirium are missed, when a patient is coded for delirium, it very likely they did have delirium. Furthermore, the addition of the use of typical and atypical antipsychotic drugs has also been shown in previous investigations to have a 99% specificity and 92% positive predictive value to identify delirium when validated against a Confusion Assessment method in postoperative patients [28]. Additionally, the combination of ICD-10 coding for delirium and the use of antipsychotic medications (as we have undertaken in our trial) when assessing delirium in postoperative patients improves the overall sensitivity. In this regard, the incidence of delirium in this study is consistent with other reports in similar settings [13, 29]. Furthermore, the ICD-coded assessment of delirium was blinded to treatment, thus unlikely to be biased between the patients. Fourth, we did not assess the severity of POD. However, we did include the need for antipsychotic medications administration, the number of doses administered, and the total dose administered, an indirect measure of severity. The inclusion of the use of antipsychotic prescription would favour the identification of a hyperactive form rather than a hypoactive forms of delirium. Thus a significant limitation of our study is that hypoactive forms of delirum may have been poorly identified. We also acknowledge that such pharmacologic treatment is only indicated when non pharmacologic strategies have been unsuccessful. However, we suggest that practically in this instance the use of pharmacologic strategies remains a common treatment in the management of hyperactive POD. We also acknowledge however, that the use of pharmacologic treatment to identify hyperactive delirium may itself be misleading particularly as postoperative agitation may occur due to other reasons (such as anxiety and postoperative pain) and may not always be associated with delirium itself. Fifth, given the post-hoc nature of this study we were unable to assess preoperative cognitive function. However, such assessments can be complex to interpret [30–32], and the randomized nature of the trial would have achieved balance for this feature. Regardless, we acknowledge it is still important to consider all such limitations with regard to the identification of POD when interpreting the results of this study. In this study, ICD-10 coded delirium was assessed for the entire duration of hospital stay and we did not collect data its timing relative to the date of surgery. However, the average hospital length of stay was only 8 days. Given this was the case for both trial groups we do not believe this would be a likely source of bias. Sixth, the difference in PaCO2 between groups could be considered small and furthermore a mean PaCO2 of 46mmHG would be consistent with only mild hypercapnia. However, the hypothesis of the study was that low tidal volume ventilation due to a higher PaCO_2_ may lead to differences in post-operative cognitive function by virtue of its physiologic intracranial effects on cerebral blood flow, cerebral metabolic rate, and intracranial pressure. Physiologically, cerebral blood flow will increase linearly in the range between 20–80 mmHg by approximately 4% per mmHg increase in PaCO_2_ [2, 3] This suggests that a difference of 6 mmHg may result in as much as a 24% difference in cerebral blood flow. However, the clinical impact of such a change is unclear and our study intended to be hypothesis generating. Finally, we did not specifically collect data on the use of potentially confounding medications such as prophylactic antibiotics of which some classes may be associated with delirium. However, given the randomized nature of the trial and similar demographics characteristics in both groups (including type of surgery), we do not believe this would be unevenly distributed and therefore would not be a likely source of bias.

## Conclusion

In this post-hoc analysis of a large randomized controlled trial, intraoperative low V_T_ compared with conventional V_T_ ventilation was not associated with an increased risk of ICD-10 coded delirium and/or the use of antipsychotic medications in adult patients undergoing major surgery.

## Supplementary Information

**Additional file 1: eTable 1. **Primary and secondary outcomes. **eTable 2. **Baseline characteristics of the included patients according to the type of surgery. **eFigure 1. **Distribution of PaCO2 Levels According to Allocation Group or Development of Postoperative Delirium.

## Data Availability

We accept participation in a data sharing arrangement on reasonable request where specifically relevant to the results of this study. These can be available from the corresponding author on reasonable request.
