# Supplementary material for: Intraoperative low tidal volume ventilation and the risk of ICD-10 coded delirium and the use for antipsychotic medications
Source: BMC Anesthesiol. 2022 May 16;22:149. doi: 10.1186/s12871-022-01689-3 (PMC9109306; doi:10.1186/s12871-022-01689-3)
Supplement: Supplementary file 1 — Additional file 1: eTable 1. Primary and secondary outcomes. eTable 2. Baseline characteristics of the included patients according to the type of surgery. eFigure 1. Distribution of PaCO2 Levels According to Allocation Group or Development of Postoperative Delirium. [file 12871_2022_1689_MOESM1_ESM.docx]

**Intraoperative Low Tidal Volume Ventilation and the Risk of Post-operative Delirium**

| **eTable 1 – Primary and secondary outcomes** | | | |
| --- | --- | --- | --- |
|  | **Low Tidal Volume**  **(*n* = 614)** | **Conventional Tidal Volume**  **(*n* = 592)** | ***p* value** |
| **Primary outcome (original trial)** |  |  |  |
| Composite respiratory complications within seven days | 231 / 608 (38.0) | 232 / 590 (39.3) | 0.636 |
| Components of the primary outcome |  |  |  |
| Pneumonia | 19 / 610 (3.1) | 22 / 591 (3.7) | 0.561 |
| Respiratory failure | 103 / 612 (16.8) | 109 / 590 (18.5) | 0.454 |
| Pleural effusion | 67 / 611 (11.0) | 69 / 590 (11.7) | 0.690 |
| Atelectasis | 150 / 608 (24.7) | 147 / 591 (24.9) | 0.935 |
| Pneumothorax | 2 / 611 (0.3) | 0 / 591 (0.0) | 0.258 |
| Bronchospasm | 5 / 612 (0.8) | 5 / 590 (0.8) | 0.954 |
| Pulmonary congestion | 10 / 612 (1.6) | 14 / 591 (2.4) | 0.362 |
| Unplanned non-invasive or invasive ventilation | 15 / 612 (2.5) | 14 / 591 (2.4) | 0.925 |
| **Secondary outcomes (original trial)** |  |  |  |
| Composite respiratory complications during hospital stay | 237 / 598 (39.6) | 239 / 579 (41.3) | 0.565 |
| Pulmonary embolism | 5 / 595 (0.8) | 6 / 573 (1.0) | 0.714 |
| Acute respiratory distress syndrome | 3 / 594 (0.5) | 0 / 574 (0.0) | --- |
| SIRS | 6 / 612 (1.0) | 13 / 591 (2.2) | 0.089 |
| Sepsis | 20 / 612 (3.3) | 13 / 591 (2.2) | 0.256 |
| Acute kidney injury | 36 / 420 (8.6) | 42 / 394 (10.7) | 0.311 |
| Risk | 24 / 36 (66.7) | 32 / 42 (76.2) |  |
| Injury | 8 / 36 (22.2) | 4 / 42 (9.5) |  |
| Failure | 4 / 36 (11.1) | 6 / 42 (14.3) |  |
| Wound infection | 11 / 597 (1.8) | 10 / 575 (1.7) | 0.893 |
| Intraoperative need of vasopressor | 518 / 600 (86.3) | 511 / 584 (87.5) | 0.551 |
| Unplanned ICU admission | 30 / 595 (5.0) | 25 / 577 (4.3) | 0.565 |
| Need for MET call | 67 / 612 (10.9) | 63 / 591 (10.7) | 0.872 |
| Length of stay |  |  |  |
| In ICU, days | 0.5 ± 1.3 | 0.6 ± 3.5 | 0.841 |
| In hospital, days | 8.3 ± 8.6 | 7.9 ± 10.6 | 0.397 |
| In–hospital mortality | 8 (1.3) | 7 (1.2) | 0.855 |
| Data are presented as mean ± standard deviation or N (%)  *ICU: intensive care unit; MET: medical emergency team* | | | |

| **eTable 2 – Baseline characteristics of the included patients according to the type of surgery** | | | | | |
| --- | --- | --- | --- | --- | --- |
|  | **Laparoscopic Surgery** | | **Open Surgery** | |  |
|  | **Low Tidal Volume**  **(*n* = 158)** | **Conventional Tidal Volume**  **(*n* = 170)** | **Low Tidal Volume**  **(*n* = 456)** | **Conventional Tidal Volume**  **(*n* = 422)** | ***p* value** |
| Age, years | 63.0 (56.0 – 71.0) | 62.0 (55.0 – 71.0) | 65.0 (54.0 – 73.0) | 65.0 (55.8 – 73.0) | 0.621 |
| Male gender | 99 (62.7) | 99 (58.2) | 267 (58.6) | 247 (58.5) | 0.806 |
| Body weight, kg |  |  |  |  |  |
| Actual | 81.0 (68.0 – 97.0) | 80.5 (71.8 – 95.8) | 80.0 (68.0 – 94.3) | 80.5 (70.0 – 92.0) | 0.697 |
| Predicted | 64.2 (57.0 – 71.3) | 63.3 (54.7 – 71.5) | 63.3 (55.1 – 70.6) | 64.2 (55.1 – 70.6) | 0.576 |
| Body mass index, kg/m^2^ | 28.1 (24.2 – 32.6) | 28.1 (24.8 – 32.3) | 27.7 (24.5 – 32.4) | 28.1 (25.1 – 32.0) | 0.905 |
| ARISCAT risk score | 31.0 (26.0 – 41.0) | 34.0 (23.0 – 41.0) | 26.0 (19.0 – 34.0) | 26.0 (19.0 – 34.0) | < 0.001 |
| Low | 25 (21.0) | 35 (28.9) | 168 (38.6) | 161 (40.6) | 0.003 |
| Moderate | 84 (70.6) | 74 (61.2) | 240 (55.2) | 208 (52.4) |  |
| High | 10 (8.4) | 12 (9.9) | 27 (6.2) | 28 (7.1) |  |
| Preoperative SpO_2_, % | 97.0 (96.0 – 98.0) | 97.0 (96.0 – 98.0) | 97.0 (96.0 – 98.0) | 97.0 (96.0 – 98.0) | 0.581 |
| Preoperative HCO_3_, mmol/L | 26.0 (24.0 – 28.0) | 26.0 (25.0 – 28.0) | 26.0 (24.0 – 27.0) | 26.0 (24.0 – 27.0) | 0.146 |
| Preoperative haemoglobin, g/dL | 141.0 (127.0 – 152.0) | 138.0 (122.0 – 149.0) | 137.0 (126.0 – 148.0) | 138.0 (124.0 – 149.0) | 0.316 |
| Preoperative creatinine, mg/dL | 0.9 (0.7 – 1.1) | 0.9 (0.7 – 1.1) | 0.9 (0.7 – 1.1) | 0.9 (0.8 – 1.1) | 0.969 |
| Co-morbidities |  |  |  |  |  |
| Diabetes mellitus | 28 (17.7) | 35 (20.6) | 91 (20.0) | 91 (21.6) | 0.782 |
| Hypertension | 81 (51.3) | 89 (52.4) | 220 (48.4) | 238 (56.5) | 0.115 |
| Coronary artery disease | 24 (15.2) | 30 (17.6) | 69 (15.2) | 70 (16.6) | 0.851 |
| Chronic renal disease | 12 (7.6) | 16 (9.4) | 44 (9.7) | 51 (12.1) | 0.402 |
| Chronic liver disease | 9 (5.7) | 19 (11.2) | 39 (8.6) | 33 (7.8) | 0.340 |
| Current smoker | 33 (20.9) | 25 (14.7) | 67 (14.7) | 84 (19.9) | 0.094 |
| COPD | 20 (12.7) | 19 (11.2) | 42 (9.2) | 46 (10.9) | 0.598 |
| Asthma | 10 (6.3) | 18 (10.6) | 56 (12.3) | 50 (11.8) | 0.188 |
| Interstitial lung disease | 0 (0.0) | 0 (0.0) | 8 (1.8) | 2 (0.5) | 0.077 |
| Bronchiectasis | 0 (0.0) | 0 (0.0) | 1 (0.2) | 1 (0.2) | 1.000 |
| Obstructive sleep apnea | 16 (10.1) | 22 (12.9) | 43 (9.4) | 41 (9.7) | 0.605 |
| Obesity* | 55 (35.9) | 68 (40.7) | 170 (38.5) | 139 (35.2) | 0.580 |
| Recent LRTI | 5 (3.2) | 1 (0.6) | 3 (0.7) | 7 (1.7) | 0.086 |
| Type of Surgery |  |  |  |  | < 0.001 |
| Abdominal | 158 (100.0) | 170 (100.0) | 190 (41.7) | 163 (38.7) |  |
| General | 0 (0.0) | 0 (0.0) | 6 (1.3) | 2 (0.5) |  |
| Ear, nose and throat | 0 (0.0) | 0 (0.0) | 17 (3.7) | 13 (3.1) |  |
| Orthopedic | 0 (0.0) | 0 (0.0) | 43 (9.4) | 46 (10.9) |  |
| Plastic | 0 (0.0) | 0 (0.0) | 31 (6.8) | 36 (8.6) |  |
| Spine | 0 (0.0) | 0 (0.0) | 125 (27.4) | 120 (28.5) |  |
| Vascular | 0 (0.0) | 0 (0.0) | 29 (6.4) | 28 (6.7) |  |
| Others | 0 (0.0) | 0 (0.0) | 15 (3.3) | 13 (3.1) |  |
| Duration of surgery, minutes | 190.0 (145.0 – 240.0) | 184.0 (134.8 – 237.8) | 189.0 (131.5 – 296.5) | 187.0 (141.0 – 251.5) | 0.291 |
| Data are presented as median (quartile 25 - quartile 75) or N (%)  *ARISCAT: Assess Respiratory Risk in Surgical Patients in Catalonia; COPD: chronic obstructive pulmonary disease; HCO_3_: bicarbonate; LRTI: lower respiratory*  *tract infection; SpO_2_: pulse oximetry*  * defined as BMI > 30 kg/m^2^ | | | | | |

**eFigure 1 - Distribution of PaCO_2_ Levels According to Allocation Group or Development of Postoperative Delirium**

**
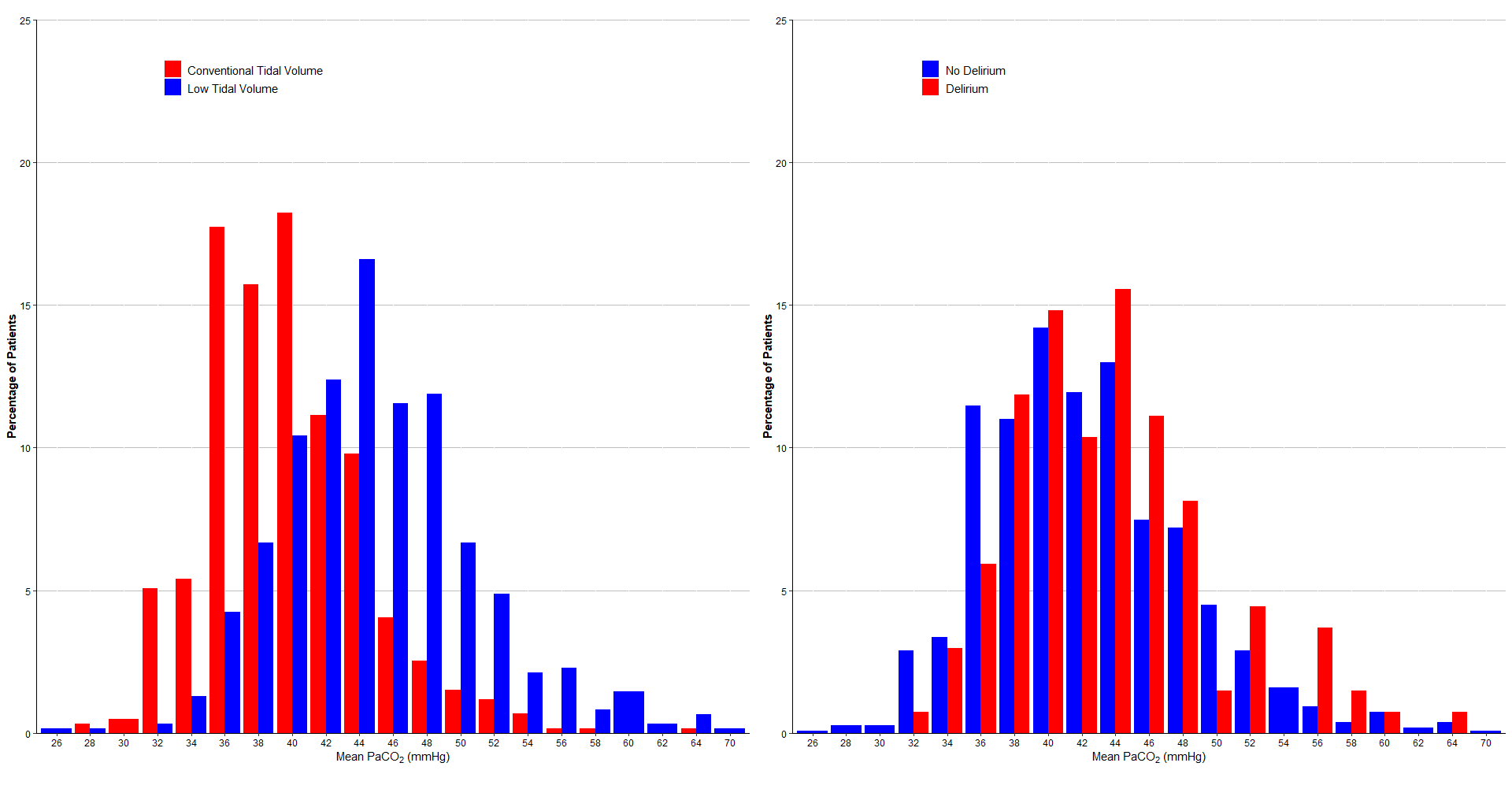
**
